# Bronchogenic cyst of the interatrial septum

**DOI:** 10.1186/1749-8090-8-171

**Published:** 2013-07-09

**Authors:** Hui Jiang, HuiShan Wang, HaiBo Wu, XinMin Li

**Affiliations:** 1 Department: Cardiovascular surgery, The Northern Hospital of Shenyang, No.83, Wenhua Road, Shenhe District, Shenyang, Liaoning Province 110016, China

**Keywords:** Congenital anomaly, Bronchogenic cyst, Interatrial septum, Diagnosis, Therapy

## Abstract

Bronchogenic cyst is considered as an uncommon congenital anomaly. It can be mostly found in mediastinum or lung. Intracardiac bronchogenic cyst is very rare. We found 2 cases in more than 20000 cardiac surgical cases in our department. The 2 cases bronchogenic cyst arose from interatrial septum (IAS), the preoperative diagnosis were myxoma, but the histological diagnosis were bronchogenic cyst in both cases. Although it is very rare, it should be considered while intracardiac tumor is diagnosed. It is recommended to complete resection of any bronchogenic cyst for primarily diagnostic and potentially therapeutic reasons, and every effort should be made to prevent complications due to injury to nearby tissues.

## Background

Bronchogenic cyst is considered as an uncommon congenital anomaly. It can be mostly found in mediastinum or lung. Intracardiac bronchogenic cyst is very rare. We found 2 cases in more than 20000 cardiac surgical cases in our department. The 2 cases bronchogenic cyst arose from interatrial septum (IAS), the preoperative diagnosis were myxoma, but the histological diagnosis were bronchogenic cyst in both cases. It is always misdiagnosed as the other intracardiac tumor.

## Case presentation

### Case 1

**Figure 1:**
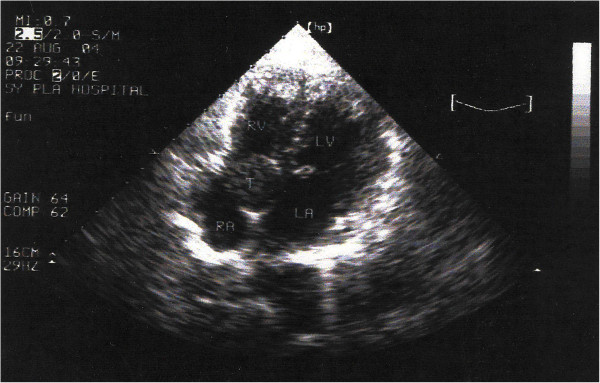
**Transthoracic echocardiography showing a 3.2 × 2.7 cm mass in the RA attached to interatrial septum.** (*LA* = left atrium; *LV* = left ventricle; *RA* = right atrium; *RV* = right ventricle; *T* = tumor).

**Figure 2:**
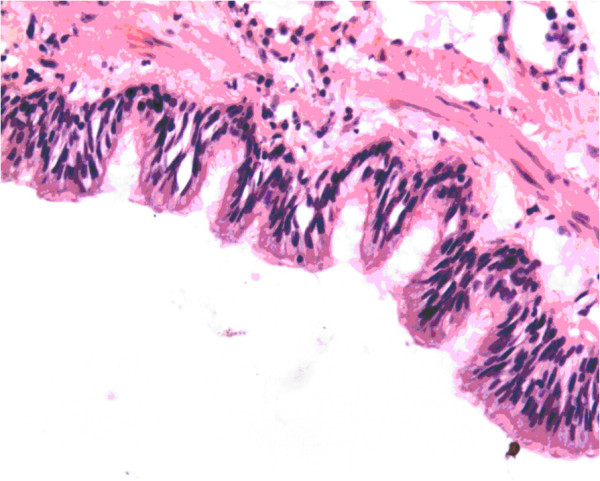
**Cyst lined by ciliated columnar epithelium, the wall contains areas of fibroelastic tissue, smooth muscle cells.** (Hematoxylin-eosin stain × 200).

A 36-year-old woman with a short history of palpitation and mild dyspnea on exertion was admitted into our clinic, physical examinations and the chest radiograph were within normal ranges, echocardiography showed the ventricular and valve function were normal, a mass of 3.2 × 2.7 cm attached to the IAS was discovered in the right atrium (Figure 1). The preoperative diagnosis was myxoma. The decision was made to resect the tumor. Under a median sternotomy and a standard cardiopulmonary bypass with cold blood cardioplegia, the right atrium was opened, a round cyst with a diameter of 3 cm arising from IAS was revealed, a 2.5 cm atrial septum defect was incidentally found behind the tumor, the lumen of the cyst contained yellow, jelly-like fluid, the cyst was resected completely after the fluid was sucked away, the defect was closed together with the ASD directly with 5/0 Prolene suture. Weaning from extracorporeal circulation was uneventful, the patient was discharged regularly on the 10^th^ day postoperative. No recurrence of the tumor was noted during the 5 year follow-up. Under microscopy, the cyst lining was ciliated columnar epithelium or so-called respiratory epithelium (Figure 2). Immunohistochemistry study showing CK (+)、Vim (+)、actin (−)、s-100 (−). Histology diagnosis was bronchogenic cyst in the interatrial septum.

### Case 2

**Figure 3:**
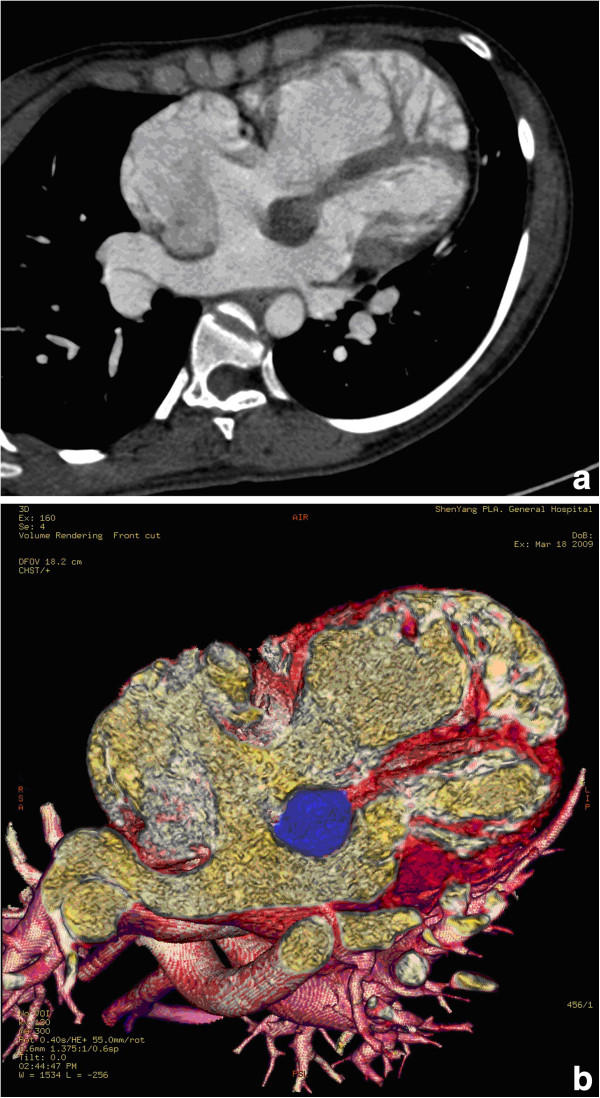
a,b Chest CT showing a 2.5 cm ASD, a 2.0 × 1.9 cm mass projecting into the left atrium, the average attenuation value was approximately 15 Hu.

**Figure 4:**
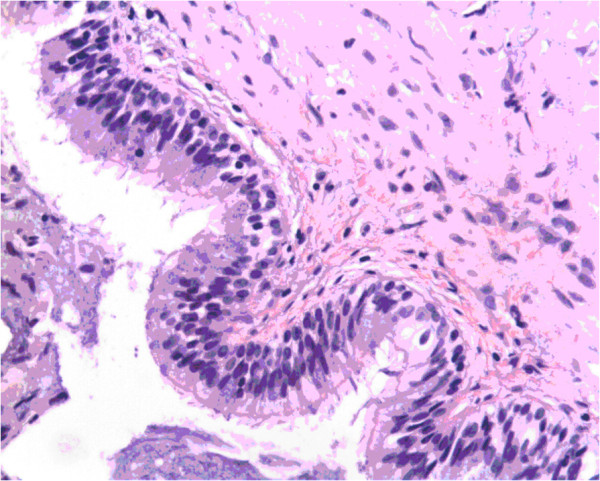
Hematoxylin-eosin stained photomicrograph (magnification × 200) showing the cyst lining of ciliated columnar epithelium with interspersed goblet cells and surrounding fibrous connective tissue.

Another 29-year-old woman with a history of palpitation and dyspnea on exertion in the last 4 months was referred to us. Systolic murmur was found by auscultation, the chest radiograph was also within normal ranges, echocardiography revealed a 2.5 cm ASD together with a 1.8 × 1.9 cm mass attached to the IAS protruding into the left atrium. Computed Tomography showing a 2.5 cm ASD, a cyst of 2.0 × 1.9 cm was found, the average attenuation value inside the left atria tumor was approximately 15 Hu (Figure 3). The operation was performed similar as the previous one. The ASD was 2.5 cm of diameter, a 2.0 cm round cyst was incised completely. The defect together with the ASD was repaired with autologous pericardium. Annuloplasty was conducted at the commissure of the posterior and septal cusp due to mild tricuspid regurgitation. Macroscopically, the cyst consisted of a 0.2 cm wall. The lumen was filled with yellow, jelly-like fluid. Microscopically, the cyst was lined with ciliated columnar epithelium, resembling ciliated respiratory epithelium, and the cyst wall was composed of fibrous connective tissue (Figure 4). Immunohistochemistry study showed CK (+)、EMA( + )、Vim (+)、s-100 (−)、CD68(-).

Postoperative course was uneventful, the patient was discharged 10 days post-operation. Echocardiography revealed no recurrence during the 3 years follow-up.

## Discussion

The location of the bronchogenic cyst can be explained by embryogenesis. Bronchogenic cysts are believed to represent a localized portion of the tracheobronchial tree that becomes separated from normal airways during the branching process and does not undergo further development. Most probably develop between the 26^th^ and the 40^th^ day of intrauterine life, during the most active period of airway development. Cardiac primordia exist in a place very near to the foregut or primitive tracheobronchial tree. At this time, abnormal budding of the tracheobronchial tree may migrate to a myocardial location; bronchogenic cysts arise from such budding. Thus, cysts are usually at the pericardium, and it is very rare that tumors are in the interatrial septum [1,2].

Intracardiac bronchogenic cysts are rare and are located on the epicardial surface or within the myocardium projecting into one of the cardiac cavities [3]. In the only review dealing with a larger number of intracardiac bronchogenic cysts, the majority were found in the right side of the heart, and only a few were located in the left side or their localization was not specified [3,4]. The location of the cyst in the two cases we reported was in the IAS, one protruding into the right atrium, the other projecting into the left atrium.

A bronchogenic cyst is a benign tumor. Many cases are asymptomatic with the lesions incidentally detected. Symptoms of intracardiac bronchogenic cysts such as chest pain, shortness of breath and arrhythmias can vary according to the location of the cyst, its size and compression of heart and vessels [1,5].

The tumor of the 1^st^ case we presented was larger than the ASD, the symptoms may come from the occlusion by the mass. But the ASD was bigger than the cyst in the 2^nd^ case, the symptoms may due to the ASD.

Most intracardiac bronchogenic cysts were found by transthoracic and transesophageal echocardiography, the diagnosis was made by contrast-enhanced chest helical CT and cardiac MRI and later was approved intraoperatively [1,6].

While considering an intracardiac tumor, myxoma, papillary fibroelastoma, thrombus, metastasis, etc. is usually considered. For a cystic tumor in the cardiac chamber, bronchogenic cyst should be in the differential diagnosis list [3,6]. The preoperative diagnosis of the two cases we reported were myxomas because bronchogenic cyst is rarely seen.

Bronchogenic cyst is a benign tumor, but definitive diagnosis should be made by pathology study. It is not possible to perform biopsy in the cardiac chamber because pulmonary or cerebral embolism may be resulted from the fluid contained. Bronchogenic cysts may develop into malignancy [7-9]. St-Georges et al. recommended all presumed bronchogenic cysts seen in the adult be resected because the majority will ultimately become symptomatic or complicated [10].

## Conclusions

Although bronchogenic cyst of interatrial septum is very rare, it should be considered while intracardiac tumor is diagnosed. It is recommended to complete resection of any bronchogenic cyst for primarily diagnostic and potentially therapeutic reasons, and every effort should be made to prevent complications due to injury to nearby tissues.

## Consent

Written informed consent was obtained from the patient for the publication of this report and any accompanying images.

## Competing interests

The authors declare that they have no competing interests.

## Authors’ contributions

HJ conceived, designed and drafted the manuscript, and performed the surgery. HSW involved in the clinical discussion of the cases. HBW was the consultant in charge of the patient’s care. XML performed the surgery and collected the imaging data. All authors read and approved the final manuscript.
